# Transection of Gut Loop Due to Post-Operative Adhesions

**Published:** 2013-05-01

**Authors:** Naeem Liaqat, Sajid Hameed Dar

**Affiliations:** Department of Paediatric Surgery, Services Institute of Medical Sciences Lahore, Pakistan

**Keywords:** Transection of gut, Adhesive bowel disease, Intestinal obstruction

## Abstract

Transection of gut due to adhesive band is an unusual complication of adhesive bowel disease. A 2-year old female presented with signs and symptoms of intestinal obstruction. Eight months earlier she underwent laparotomy for excision of duplication cyst of ileum. Exploratory laparotomy performed during current admission showed complete transection of gut.

## INTRODUCTION

Intestinal obstruction secondary to adhesions after surgical procedure is not an uncommon entity and is routinely encountered. Due to adhesive bands, complete transection of gut loop is an entity that has not yet been reported in literature. A case of complete transection of gut loop secondary to adhesive bowel disease is being reported.

## CASE REPORT

A 2-year old female presented with recurrent pain abdomen and non-bilious vomitting. At the age of 18 months she was operated for duplication cyst of ileum which was located 6 cm proximal to ileocecal junction. During the last eight months she had multiple episodes of adhesive intestinal obstruction responding well to conservative treatment. During last episode she responded initially but symptoms recurred. Her barium follow through study performed showed grossly distended small intestine with a cutoff point beyond which no contrast passed. On exploration, two blind ending loops, lying close to each other (Fig. 1), 15cm distal to DJ junction found. Proximal loop was grossly dilated while distal loop was collapsed. Both loops showed signs of scarring. End to end anastomosis was done. Postoperative recovery was uneventful. She is doing fine at eight months follow up.

**Figure:**
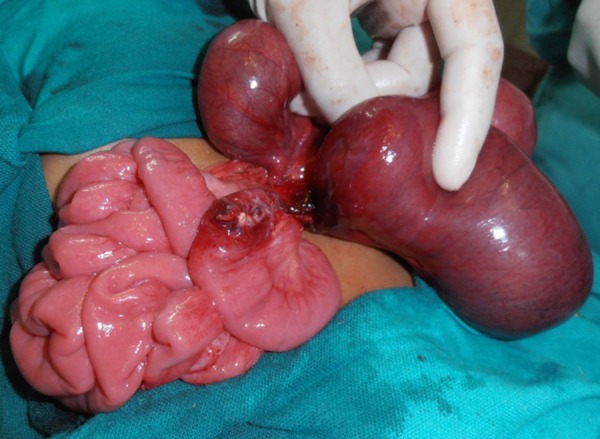
Figure 1: Showing two blind ends of the bowel.

## DISCUSSION

Adhesive bowel disease is an important cause of intestinal obstruction and comprises of 65-75% of all small bowel obstructions [1]. Usually these adhesions respond to conservative management but intervention is required in 15-18% of cases [2]. Any source of peritoneal irritation can result in local fibrin production which may lead to adhesion formation. Most common sites of adhesion are that of anastomosis and raw areas left after attempts at reperitonealization of serosal tear. In addition, presence of trauma, talc, gauze and silk suture predispose to adhesion formation [1]. The adhesions may form a tight band that may ultimately lead to complete transection leading to the formation of two blind loops as happened in our case.

## Footnotes

**Source of Support:** Nil

**Conflict of Interest:** None declared

